# Outcomes by best response with hypomethylating agent plus venetoclax in adults with previously untreated acute myeloid leukemia

**DOI:** 10.1007/s00277-024-05976-6

**Published:** 2024-09-07

**Authors:** Akriti G. Jain, Virginia O. Volpe, Chen Wang, Somedeb Ball, Katherine Tobon, Onyee Chan, Eric Padron, Andrew Kuykendall, Jeffrey E. Lancet, Rami Komrokji, David A. Sallman, Kendra L. Sweet

**Affiliations:** 1 Leukemia and Myeloid Disorders , Taussig Cancer Institute, Cleveland Clinic, Cleveland, OH USA; 2 Department of Malignant Hematology, Dana Farber Cancer Institute, Boston, MA USA; 3 Department of Internal Medicine, Morsani College of Medicine, University of South Florida, Tampa, FL USA; 4 Vanderbilt-Ingram Cancer Center, Nashville, TN USA; 5 Department of Malignant Hematology, H. Lee Moffitt Cancer Center and Research Institute, Tampa, FL USA

**Keywords:** Complete remission, MLFS, Morphological leukemia free state, CRi, ELN 2022, AML, CR

## Abstract

Introduction: We aimed to compare outcomes of patients with AML treated with frontline hypomethylating agent and venetoclax (HMA + Ven) who achieved complete remission (CR), complete remission with partial hematologic recovery (CRh), complete remission with incomplete hematologic recovery (CRi), or morphologic leukemia-free state (MLFS) as defined by ELN 2022. Methods: Patients with AML seen at Moffitt Cancer Center between 2018 and 2022 and treated with HMA + Ven were retrospectively evaluated. Results: About 120 patients achieved blast clearance with best response of CR in 52 (43.3%), CRh in 22 (18.3%), CRi in 31 (25.8%) and MLFS in 15 (12.5%) patients. Greater proportion of patients with MLFS had a prior myeloid malignancy (*p* = 0.003) and were treated with prior HMA (*p* < 0.001). Patients that achieved MLFS as their best response had inferior OS compared to the CR/CRh/CRi cohort (8 months vs. 27 months; *p* < 0.001). RFS was also worse for the MLFS cohort. Conclusion: To the best of our knowledge, this is the largest study analyzing differences in outcomes of AML patients treated with HMA + Ven based on best response. We noted that prior myeloid malignancy and use of HMA led to more MLFS as best response compared to CR/CRi. The OS and RFS were inferior for MLFS cohort.

## Introduction

Acute myeloid leukemia (AML) is a heterogenous, clonal hematopoietic malignancy with an aggressive clinical course and poor outcomes. Our understanding of its disease biology is improving with each passing decade. We have come a long way from the standard, one size fits all, “7 + 3” induction chemotherapy approach introduced in 1973 to a more individualized, patient centric approach focusing not only on disease control but also quality of life [1]. The standardized curative approach of induction chemotherapy followed by consolidation chemotherapy and then allogeneic stem cell transplantation (alloSCT) is not always an option in patients with advanced age and/or pre-existing conditions. In such cases non-intensive approaches including hypomethylating agents (HMA) or low dose cytarabine (LDAC) are being used. In addition, there are many emerging and established targets such as IDH1, IDH2, FLT3 and CD33 that have led to development of effective small molecule strategies for treatment of AML. B-cell leukemia/lymphoma (BCL-2), an antiapoptotic protein, is one such target which is overexpressed in hematological malignancies [2]. It has been associated with maintenance and survival of AML cells, resistance to treatments, and hence poor outcomes in patients with AML [2]. Venetoclax is a selective, small molecule BCL-2 inhibitor. The pivotal phase III VIALE-A trial led to approval of venetoclax in combination with azacitidine for treatment of newly diagnosed AML in patients aged 75 or older, or those ineligible for intensive chemotherapy [3]. From a 30% incidence of remission and an overall survival (OS) of less than a year with single agent HMA, the combination of azacitidine and venetoclax (HMA + Ven) led to a 66.4% composite complete remission rate (CCR) with a 14.7 month OS [3–5].

The response criteria used in VIALE-A used a combination of complete remission (CR) and complete remission with incomplete count recovery (CRi) to calculate the CCR. However, the true CR rate was only 36.7% in the HMA + Ven arm with approximately half of the remissions being CRi. The rate of complete remission with partial hematologic recovery (CRh) was a secondary endpoint of this trial. Morphologic leukemia free state (MLFS) was not a response criteria defined by investigators in VIALE-A, however it is used in many other clinical trials and often in standard practice. Given that cytopenias caused by myelosuppression is a significant side effect of HMA + Ven and many of the responses seen with this regimen are either CRi, CRh, or MLFS, the aim of our study was to determine the prognostic impact of the type of best response (CR, CRi or MLFS) in patients with newly diagnosed AML treated with HMA + Ven.

## Methods

### Study subjects

This is a retrospective study conducted at Moffitt Cancer Center from a database of 200 patients treated with HMA + Ven for previously untreated AML between 2018 and 2022. For this analysis, we included 120 patients who achieved bone marrow blast clearance < 5% (CR/CRi/CRh/MLFS) after treatment with HMA + Ven. Chart review was performed to collect patient baseline characteristics, pathology reports including cytogenetics and next generation sequencing results, treatment details including number of cycles of HMA + Ven, alloSCT, and treatment outcomes such as relapse free survival (RFS) and overall survival (OS). The study was approved by the institutional review board at Moffitt Cancer Center.

### Definitions and response criteria

AML subtypes were categorized according to the 2016 WHO classification [6]. Response, relapse, and genetic risk categories were defined according to the 2022 European Leukemia Net (ELN) criteria [7]. Complete remission (CR) was defined as an absolute neutrophil count ≥ 1.0 × 10^9^/L, a platelet count ≥ 100 × 10^9^/L, red-cell transfusion independence, and < 5% blasts in the bone marrow. Complete remission with partial hematologic recovery (CRh) was defined as all the criteria for CR, except that both the neutrophil and platelet counts were lower than the threshold designated for complete recovery (for neutropenia ≥ 0.5 × 10^9^/L and a platelet count of more than ≥ 50 × 10^9^/). Complete remission with incomplete hematologic recovery (CRi) was defined as all the criteria for CR, except for neutropenia (absolute neutrophil count, < 1.0 × 10^9^/L) or thrombocytopenia (platelet count, < 100 × 10^9^/L). Morphologic leukemia free state (MLFS) was defined as bone marrow blasts < 5% with an at least 10% cellularity in the marrow; absence of circulating blasts and no hematologic recovery was required. As per ELN 2022, response definitions for patients with marrow blast clearance (< 5%) were adjusted to reflect the best hematologic response achieved prior to commencement of the next treatment cycle. Aspirate reports that include MLFS, CRh, or CRi were altered based on post-marrow blood counts for the final response designation. Since HMA + Ven is considered a non-intensive treatment option, best response was designated within 180 days of initiation of treatment as defined by ELN 2022. Genetic mutation analyses was performed using next-generation sequencing (NGS) with TruSeq myeloid gene set or a TruSight myeloid panel. OS for the entire cohort was calculated as the time from the date of AML diagnosis to death from any cause or date of last follow-up. RFS was calculated from date of best response to date of relapse/death or last follow up whichever occurred first.

### Statistical analyses

For categorical variables, frequencies differences were evaluated using Chi-square test. The Kruskal-Wallis test was used for comparing continuous variables. Survival curves were calculated using the Kaplan–Meier method and log-rank tests were applied for univariate comparisons. In all analyses, a p-value < 0.05 was considered significant. Statistical analyses were performed using GraphPad Prism^®^ 7 (GraphPad Software Inc., La Jolla, USA) and IBM SPSS Statistics (SPSS Inc. Chicago, IL).

## Results

A total of 200 patients received HMA + Ven as treatment for their newly diagnosed AML. Of these 200 patients, 120 (60%) achieved blast clearance at any time with best response of CR in 52 (43.3%), CRh in 22 (18.3%), CRi in 31 (25.8%) and MLFS in 15 (12.5%) patients. Table 1 shows the baseline characteristics of all 120 patients included in this analysis. The median age at diagnosis was 75 years (range 41–89). The majority of the patients were male (*n* = 80, 66.7%) and Caucasian (*n* = 110, 91.7%). While most patients had an ECOG performance status of 1 (*n* = 93; 77.5%), a minority had an ECOG PS of 2 or 3 (*n* = 8, 6.7% and *n* = 2, 1.7% respectively). Twenty-seven patients (22.5%) had a history of antecedent hematologic malignancy, however only 10 (8.3%) received treatment with an HMA prior to progression to AML. Thirty-four patients (28.3%) had a prior non-myeloid hematological malignancy such as lymphoma or a solid tumor. The baseline laboratory characteristics are described in Table 1. Two patients in this cohort had extramedullary AML. AML with myelodysplasia related changes (AML-MRC) was the most common AML WHO diagnosis (*n* = 61, 50.8%) followed by AML not otherwise specified (NOS; *n* = 43, 35.8%). Whilst most patients’ disease was characterized as intermediate cytogenetic risk (*n* = 80, 70.2%), when molecular data was incorporated to determine the ELN 2022 risk stratification, the majority of patients were classified as adverse risk (*n* = 70, 58.3%).

**Table 1:** Baseline characteristics of all the patients included in the analysis

Variable		All, *n* (%) *n* = 120
**Age**,** years**,** median (range)**		75 (41–89)
**Gender**,** n (%)**		
	Male	80 (66.7)
	Female	40 (33.3)
**Race**,** n (%)**		
	White	110 (91.7)
	Black	4 (3.3)
	Hispanic	1 (0.8)
	Asian	1 (0.8)
	Other	4 (3.3)
**ECOG PS**,** n (%)**		
	0	17 (14.2)
	1	93 (77.5)
	2	8 (6.7)
	3	2 (1.7)
**Prior HMA**,** n (%)**		10 (8.3)
**Laboratory Values average (median**,** range)**		
	WBC (x10^9^/L)	3.1 (0.1–36)
	ANC (x10^9^/L)	0.7 (0.01–20.8)
	Hemoglobin (g/dl)	8.8 (6.1–12.4)
	Platelets (x10^9^/L)	67 (8-706)
	Circulating Blasts (%)	12 (0–74)
**Bone Marrow Blasts (%**,** median**,** range)**		34.9 (10–95)
**Extramedullary disease**,** n (%)**		2 (1.7)
**AML WHO diagnosis**		
	AML with recurrent cyto abn	2 (1.7)
	AML-MRC	61 (50.8)
	t-AML	14 (11.7)
	AML, NOS	43 (35.8)
**Cytogenetic risk**,** n (%)**		
	Intermediate	80 (70.2)
	Adverse	34 (29.8)
**ELN 2022 risk**,** n (%)**		
	Favorable	11 (9.2)
	Intermediate	39 (32.5)
	Adverse	70 (58.3)
**Prior Non-myeloid Malignancy**,** n (%)**		34 (28.3)
**Antecedent Hematological Malignancy**,** n (%)**		27 (22.5)

Table 2 describes the differences in the baseline disease and laboratory characteristics between the four cohorts (CR, CRh, CRi and MLFS). The median age at diagnosis was similar between the four groups. There were no significant differences based on race, ECOG PS, cytogenetic risk, ELN 2022 risk or prior non-myeloid malignancy. Antecedent myeloid malignancy was seen in greater proportion in the patients who achieved MLFS as their best response compared to those who achieved a CR, CRh or CRi (*n* = 9, 60% (MLFS) vs. *n* = 9, 17.3% (CR), *n* = 3, 13.6% (CRh) and *n* = 6, 19.4% (CRi); *p* = 0.003). Similarly, those who had received prior HMA had a higher rate of MLFS as best response compared to CR, CRh or CRi (*n* = 6, 40% (MLFS) vs. *n* = 2, 3.8% (CR), *n* = 0 (CRh) and *n* = 2, 6.5% (CRi); *p* < 0.001). While there was no difference in baseline laboratory characteristics at diagnosis between the three cohorts, bone marrow blasts were significantly lower in the patients who achieved MLFS as their best response (25% (MLFS) vs. 45% (CR), 31% (CRh) and 30% (CRi); *p* = 0.045) compared to patients who attained CR, CRh and CRi as their best response.

The results of next generation sequencing identified the most common mutations seen in all patients at diagnosis to include *TET2* (*n* = 21, 26.7%), *ASXL1* (*n* = 28, 24.1%), *SRSF2* (*n* = 26, 22.4%), *RUNX1* (*n* = 26, 22.4%), *TP53* (*n* = 25, 21.6%), *DNMT3A* (*n* = 21, 18.1%), *IDH2* (*n* = 15, 12.9%) and *NPM1* (*n* = 12, 10.3%). A higher proportion of patients with MLFS as their best response harbored mutations in *TET2* and *SETBP1* although this was not significant (Table 3).

**Table 2:** Baseline characteristics according to best response

Variable		CR, *n* (%) *n* = 52 (43.3)	CRh, *n* (%) *n* = 22 (18.3)	CRi, *n* (%) *n* = 31 (25.8)	MLFS, *n* (%) *n* = 15 (12.5)	*p*-value
**Age**,** years**		75 (41–86)	76.5 (58–86)	75 (60–89)	77 (67–89)	0.238
**Gender**,** n (%)**						0.519
	Male	33 (63.5)	13 (59.1)	22 (71)	12 (80)	
	Female	19 (36.5)	9 (40.9)	9 (29)	3 (20)	
**Race**,** n (%)**						0.322
	White	49 (94.2)	19 (86.4)	29 (93.5)	13 (86.7)	
	Black	1 (1.9)	1 (4.5)	0	2 (13.3)	
	Hispanic	1 (1.9)	0	0	0	
	Asian	0	1 (4.5)	0	0	
	Other	1 (1.9)	1 (4.5)	2 (6.5)	0	
**ECOG PS**,** n (%)**						0.641
	0	9 (17.3)	2 (9.1)	5 (16.1)	1 (6.7)	
	1	41 (78.8)	17 (77.3)	23 (74.2)	12 (80)	
	2	1 (1.9)	3 (13.6)	2 (6.5)	2 (13.3)	
	3	1 (1.9)	0	1 (3.2)	0	
**Prior HMA**,** n (%)**		2 (3.8)	0	2 (6.5)	6 (40)	< 0.001
**Laboratory Values**,** median (range)**						
	WBC (x10^9^/L)	3.5 (0.6–33.9)	2.6 (0.1–27.9)	2.3 (0.4–36.0)	7.4 (1.0-32.8)	0.331
	ANC (x10^9^/L)	0.8 (0.1–15.6)	1.1 (0.1–5.5)	0.4 (0.03–20.8)	1.6 (0.01–13.5)	0.450
	Hemoglobin (g/dl)	8.4 (61.-12.4)	8.9 (6.7–10.5)	9.2 (6.3–12)	9.6 (7.3–11.7)	0.663
	Platelets (x10^9^/L)	61 (8-590)	76 (15–237)	66 (20–706)	56 (18–499)	0.861
	Circulating Blasts (%)	5.5 (0–70)	22 (5–63)	2 (0–64)	19 (5–74)	0.022
**Bone Marrow Blasts (%**,** median**,** range)**		45 (17–95)	31 (12–91)	30 (20–80)	25 (20–80)	0.045
**Extramedullary disease**,** n (%)**		2 (6.25)	0	0	0	0.447
**AML WHO diagnosis**						0.052
	AML with recurrent cyto abn	0	2 (9.1)	0	0	
	AML-MRC	25 (48.1)	8 (36.4)	16 (51.6)	12 (80)	
	t-AML	7 (13.5)	2 (9.1)	5 (16.1)	0	
	AML, NOS	20 (38.5)	10 (45.5)	10 (32.3)	3 (20)	
**Cytogenetic risk**,** n (%)**						0.671
	Intermediate	32 (65.3)	16 (80)	21 (70)	11 (73.3)	
	Adverse	17 (34.7)	4 (20)	9 (30)	4 (26.7)	
**ELN 2022 risk**,** n (%)**						0.634
	Favorable	5 (9.6)	3 (13.6)	2 (6.5)	2 (13.3)	
	Intermediate	17 (32.7)	9 (40.9)	10 (32.3)	2 (13.3)	
	Adverse	30 (57.7)	10 (45.5)	19 (61.3)	11 (73.3)	
**Prior Non-myeloid Malignancy**,** n (%)**		17 (32.7)	5 (22.7)	9 (29)	3 (20)	0.717
**Antecedent Hematological Malignancy (myeloid)**,** n (%)**		9 (17.3)	3 (13.6)	6 (19.4)	9 (60)	0.003

**Table 3:** Mutations in the three response categories

Mutation	All (*n* = 116) *n* (%)	CR, *n* (%) *n* = 52 (43.3)	CRh, *n* (%) *n* = 22 (18.3)	CRi, *n* (%) *n* = 31 (25.8)	MLFS, *n* (%) *n* = 15 (12.5)	*p*-value
**TET2**	31 (26.7)	12 (24.5)	4 (19)	7 (22.6)	8 (53.3)	0.092
**ASXL1**	28 (24.1)	12 (24.5)	2 (9.5)	9 (29)	5 (33.3)	0.314
**SRSF2**	26 (22.4)	6 (12.2)	7 (33.3)	7 (22.6)	6 (40)	0.071
**RUNX1**	26 (22.4)	13 (26.5)	2 (9.5)	6 (19.4)	5 (33.3)	0.298
**TP53**	25 (21.6)	11 (22.4)	2 (9.5)	10 (32.2)	2 (13.3)	0.210
**DNMT3A**	21 (18.1)	9 (18.4)	5 (23.8)	5 (16.1)	2 (13.3)	0.855
**IDH2**	15 (12.9)	4 (8.2)	3 (14.3)	6 (19.4)	2 (13.3)	0.539
**NPM1**	12 (10.3)	3 (6.1)	4 (19)	3 (9.7)	2 (13.3)	0.421
**NRAS**	11 (9.5)	7 (14.3)	2 (9.5)	0	2 (13.3)	0.185
**FLT3**	7 (6)	3 (6.1)	1 (4.8)	2 (6.5)	1 (6.7)	0.994
**U2AF1**	7 (6)	2 (4.1)	1 (4.8)	2 (6.5)	2 (13.3)	0.613
**PHF6**	6 (5.2)	2 (4.1)	1 (4.8)	2 (6.5)	1 (6.7)	0.960
**PTPN11**	5 (4.3)	1 (2)	2 (9.5)	1 (3.2)	1 (6.7)	0.515
**ZRSR2**	5 (4.3)	4 (8.2)	0	0	1 (6.7)	0.230
**BCOR**	5 (4.3)	0	1 (4.8)	3 (9.7)	1 (6.7)	0.205
**STAG2**	5 (4.3)	1 (2)	1 (4.8)	1 (3.2)	2 (!3.3)	0.299
**FLT3 TKD**	5 (4.3)	2 (4.1)	0	1 (3.2)	2 (13.3)	0.261
**EZH2**	4 (3.4)	2 (4.1)	1 (4.8)	0	1 (6.7)	0.628
**SETBP1**	4 (3.4)	1 (2)	1 (4.8)	1 (3.2)	1 (6.7)	0.832
**SF3B1**	4 (3.4)	3 (6.1)	0	0	1 (6.7)	0.337

**Table 4:** Differences in treatment variables and outcomes between the three cohorts

Variable		CR, *n* (%) *n* = 52	CRh, *n* (%) *n* = 22	CRi, *n* (%) *n* = 31	MLFS, *n* (%) *n* = 15	*p*-value
**AlloSCT in CR1**,** n (%)**		14 (26.9)	3 (13.6)	4 (12.9)	1 (6.7%)	0.284
**HMA**,** n (%)**						0.275
	Azacitidine	35 (67.3)	16 (72.7)	23 (74.2)	7 (46.7)	
	Decitabine	17 (32.7)	6 (27.3)	8 (25.8)	8 (53.3)	
**Relapse**,** n (%)**		20 (38.5)	11 (50)	14 (45.2)	4 (26.7)	0.498

The overall rate of alloSCT in CR1 was 18.3% (*n* = 22). There was no significant difference in the likelihood of proceeding to alloSCT in CR1 in patients who achieved CR (*n* = 14, 26.9%) compared to patients who achieved CRh, CRi and MLFS (*n* = 3; 13.6%, *n* = 4; 12.9% and *n* = 1; 6.7% respectively; *p* = 0.284). There were no significant differences in best response based on the HMA used (azacitidine or decitabine). Additionally, no significant differences were noted in relapse rate between the four cohorts (Table 4).

**Fig. 1:**
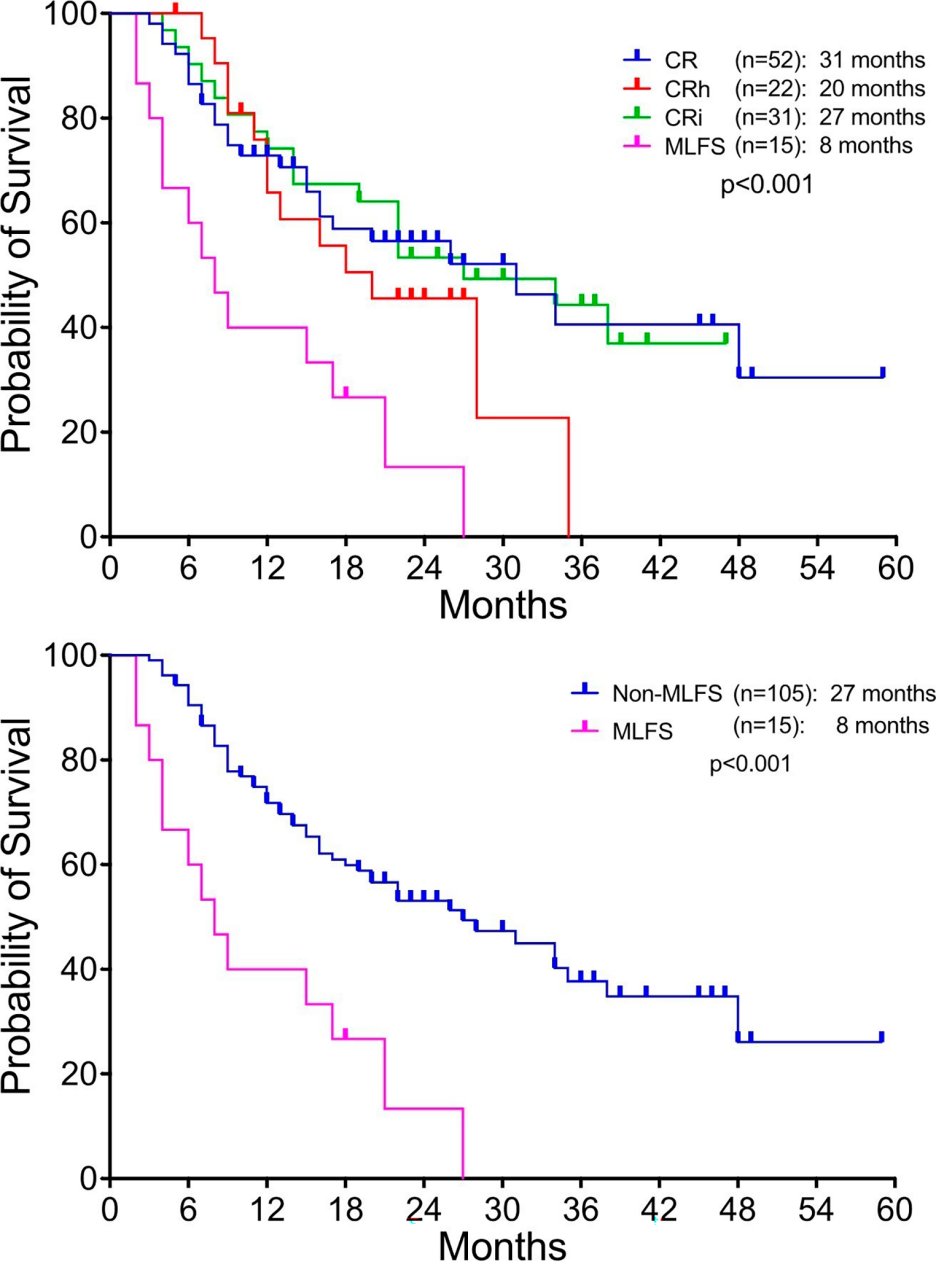
Overall survival of based on best response

**Fig. 2:**
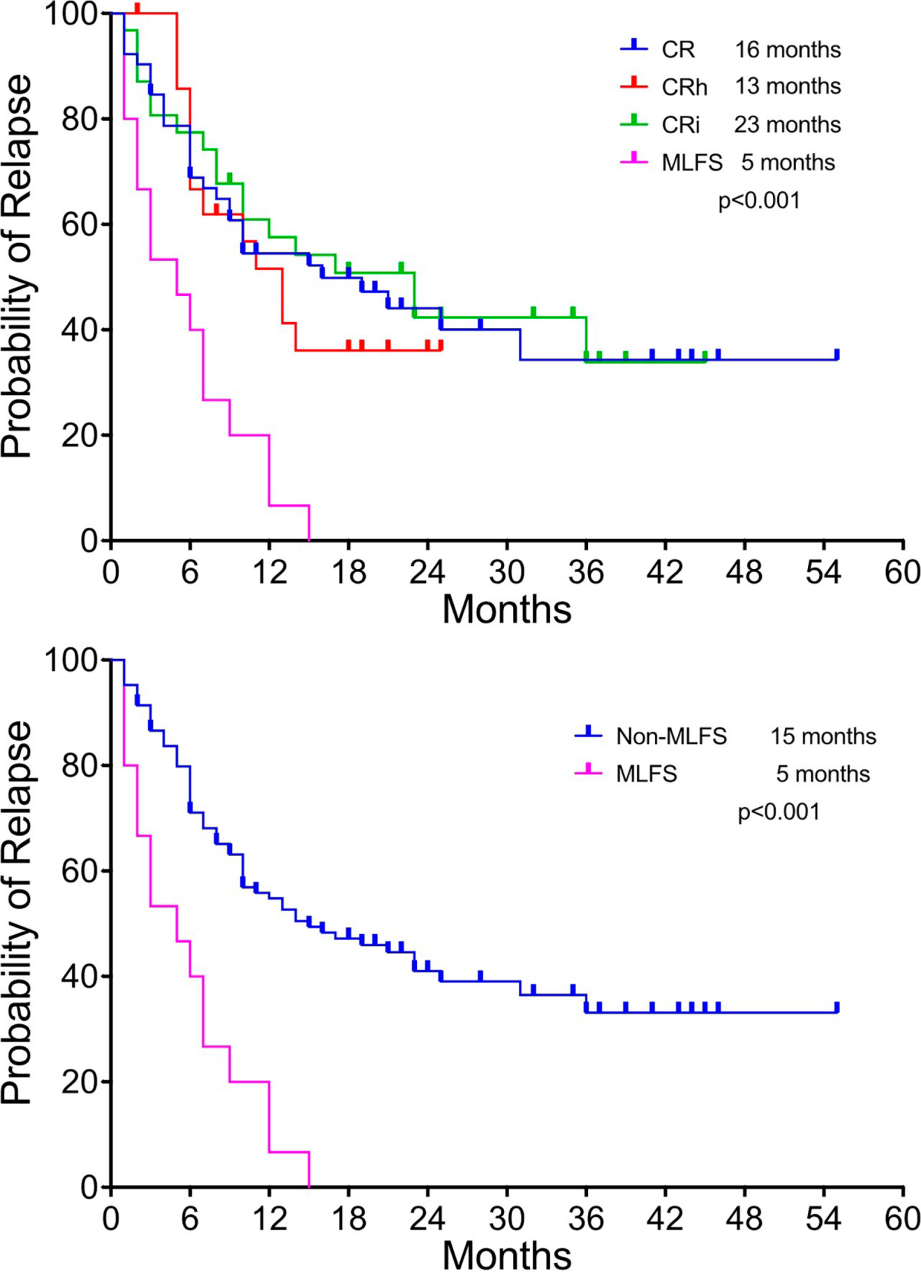
Relapse free survival based on best response

At a median follow up of 27 months, the OS for the entire cohort was 22 months. When looking at differences in OS based on best response, the OS for patients with MLFS as best response was significantly shorter compared to CR, CRh and CRi (8 vs. 31 (CR), 20 (CRh) and 27 (CRi) months; *p* < 0.001; Fig. 1). We then compared patients with MLFS as best response to the non-MLFS cohort which included patients that attained CR, CRh and CRi and the OS for patients with MLFS continued to be significantly inferior (8 vs. 27 months; *p* < 0.001). For the entire cohort, the median OS for patients who underwent alloSCT in CR1 was significantly longer compared to patients who did not (Not reached vs. 20 months; *p* = 0.004). The RFS was significantly worse for the MLFS (5 months) cohort compared to CR (16 months), CRh (13 months) or CRi (23 months) (*p* < 0.001; Fig. 2).

Serial NGS data was only available for 76 out of the 120 patients included in this study. Of these 76 patients, 9 had a negative NGS at diagnosis. Out of the remaining 67 patients, 25 (37.3%) cleared all of their mutations at the time of their documented best response and 42 (62.7%) had persistent mutations at best response. Looking at differences in clearance of mutations in the three cohorts, there was no significant difference in the number of patients that cleared mutations based on best response (CR 45.4%, *n* = 15/33, CRh: 50%; *n* = 4/8, CRi (26.3, *n* = 5/19) and MLFS (14.3%; *n* = 1/7; *p* = 0.255).

## Discussion

The less intensive approach of combining HMA with venetoclax for treatment of newly diagnosed AML has revolutionized the management of this disease in patients with advanced age and/or other pre-existing conditions which make them ineligible for intensive chemotherapy. To the best of our knowledge, this the largest study analyzing differences in outcomes of patients treated with HMA + Ven for newly diagnosed AML based on the best response achieved with the combination treatment. In this analysis there was no significant difference in OS or RFS for patients who achieved attained CR or CRi/CRh, however the patients whose best response was MLFS had significantly worse OS and RFS.

Achieving CR using the strict response criteria for hematological count recovery was an essential milestone in the treatment of AML in the era of intensive induction chemotherapy as demonstrated by Freireich et al. in the 1960s [8]. Hence for many years, CR or not was the only criteria used for assessing response in AML and was used as a proxy for superior long-term outcomes [9]. CRi as a measure of response was introduced in 2003 by the International Working Group [10]. MLFS, generally used in the context of clinical trials was introduced as a response criterion by the ELN 2017 recommendations. Most recently, CRh has emerged as another response criteria for AML. It was not officially included in the ELN 2017 guidelines but it made its way into the response criteria laid down by the ELN 2022 expert panel [7]. CR with incomplete platelet recovery (CRp) is another criterion in the studies of relapsed AML with gemtuzumab ozogamicin [11]. Consequently, CRp was adapted together with CRi.

Some retrospective studies have reported inferior OS and RFS for patients achieving CRi compared to CR. In a large study on 2,228 patients from MD Anderson Cancer Center, Walter et al. reported that patients achieving CR were more likely to be alive at 3 years, and at 5 years than patients achieving CRp (relative risk for 5-year survival, 3.0; 95% CI, 1.0 to 9.0; *P* < 0.02) [12]. In a study on 125 patients treated with decitabine and venetoclax, Siddiqui et al. showed that CRh was associated with a trend towards improved OS and EFS compared to CRi and MLFS [13]. The rates of CR, CRh, CRi and MLFS were 55% (*n* = 69), 8% (*n* = 11), 11% (*n* = 14) and 8% (*n* = 10) respectively. The median OS and EFS for patients achieving CR was 24.5 months and 18 months compared to OS of 16.9 months for CRh, 5.8 months for CRi and 6.2 months for MLFS and a EFS of 14.1 months for CRh, 5.8 months for CRi and 6.2 months for MLFS. Similarly, we noted a poor OS and RFS in patients whose best response was MLFS in our study however no differences were seen in outcomes between patients who attained CR/CRi or CRh. In addition, CRh did not lead to significantly improved outcomes compared to CRi.

Chen et al., in their study looking at minimal residual disease (MRD) in the context of best response, showed that the frequency of MRD positivity assessed by multiparameter flow cytometry (MFC) was lowest in patients achieving CR (19.0%), higher in the CRp group (54.2%), and highest in the CRi group (60.9%; *P* < 0.01). In our analysis, a numerically higher number of patients cleared mutations in the CR and CRh cohort compared to CRi and MLFS however this was not significantly higher probably due to small numbers (CR 45.4%, *n* = 15/33, CRh: 50%; *n* = 4/8, CRi (26.3, *n* = 5/19) and MLFS (14.3%; *n* = 1/7; *p* = 0.255). It is essential to note the significant difference in using MFC vs. NGS for an analysis such as this, however, given the far greater sensitivity to detect MRD with MFC.

Some interesting findings in our study include the higher rates of MLFS seen in patients with prior myeloid malignancy and pre-treatment with HMA. This could be a reflection of the disease biology and/or damaged marrow from prior treatment. However, it is important to note that there were no significant differences between the three groups based on ELN risk or cytogenetic risk that impacted outcomes. The similar outcomes seen in CR and CRi could be a function of the better understanding and dose modifications of subsequent treatment cycles to allow for count recovery. Some factors that may have affected the poor outcomes in patients with MLFS is the low rate of alloSCT in that cohort (6.7%) compared to 26.9% in patients who achieved CR, 13.6% who attained CRh and 12.9% in patients that achieved CRi. However, in the same token, patients treated with HMA + Ven are typically older and/or with co-existing conditions which make them ineligible for alloSCT to begin with.

Despite limitations in our study including inherent bias due to retrospective design, small number of patients with a best response of MLFS, lack of MRD data for all patients and outcomes related to MRD negative remissions, our study provides important conclusions on the differences in outcomes based on best response in patients treated with HMA + Ven for AML. We noted some factors which could be predictive of best response including prior myeloid malignancy and/or use of HMA which led to more MLFS responses compared to CR or CRi. Additionally, mutations in *TET2* and *SRSF2* were seen in higher proportions in patients who attained MLFS as their best response. *SRSF2* is thought to be a “secondary type” mutation resulting in outcomes similar to patients with a known prior myeloid malignancy [14]. This could partially explain the correlation between these mutations and the higher rates of MLFS similar to what is seen in the patients with known AHM. The OS and RFS was significantly inferior for the MLFS cohort while there were no differences in OS and RFS between the CR, CRh and CRi cohorts.

## Data Availability

Data can be shared on request at the discretion of the senior author.
